# Autonomic arousal profiles in adolescents and young adults with ADHD as a function of recording context

**DOI:** 10.1016/j.psychres.2019.03.039

**Published:** 2019-05

**Authors:** Ebba Du Rietz, Sarah-Naomi James, Tobias Banaschewski, Daniel Brandeis, Philip Asherson, Jonna Kuntsi

**Affiliations:** a King's College London, Social, Genetic and Developmental Psychiatry Centre, Institute of Psychiatry, Psychology and Neuroscience, 16 De Crespigny Park, Denmark Hill, London, SE5 8AF, United Kingdom; b MRC Unit for Lifelong Health and Ageing at UCL, University College London, 33 Bedford Place, London, WC1B 5JU, United Kingdom; c Department of Child and Adolescent Psychiatry and Psychotherapy, Central Institute of Mental Health, Medical Faculty Mannheim/ Heidelberg University, Square J5, 68159 Mannheim, Germany; d Department of Child and Adolescent Psychiatry, University of Zurich, Neumünsterallee 9, 8032 Zurich, Switzerland

**Keywords:** Tonic arousal, Phasic arousal, Electrodermal activity, Skin conductance, ADHD

## Abstract

A recent study (James et al. 2016) found that attention-deficit/hyperactivity disorder (ADHD) was associated with hypo-arousal, indexed by low electrodermal activity, during a low-demand reaction-time task, which normalized in a fast-incentive condition. We now investigate if (1) autonomic arousal in individuals with ADHD changes over a long testing session and (2) across time, to clarify if arousal profiles are context-dependent. We also examine (3) how autonomic arousal relates to each ADHD symptom domain, and specificity of arousal profiles to ADHD, by controlling for oppositional defiant/conduct disorder (ODD/CD) symptoms. Skin conductance level and non-specific fluctuations were measured during four successive resting-state and cognitive conditions (Resting-state time 1, Continuous Performance Task, Fast Task: Baseline and Fast-Incentive conditions, Resting-state time 2) from 71 adolescents/young adults with ADHD and 140 controls. Lower arousal was observed in individuals with ADHD only during a slow, low-demanding task, and more fluctuating arousal was observed towards the end of assessment. Both inattentive and hyperactive-impulsive symptoms were associated with arousal levels and fluctuations, independently from ODD/CD. Overall, we extend previous findings showing that under-arousal, but also fluctuating arousal, are context-specific rather than stable impairments in ADHD.

## Highlights

• Individuals with ADHD showed atypical autonomic arousal only in certain experimental conditions.
• The findings suggest that atypical arousal in ADHD does not reflect a stable impairment.
• Phasic and tonic arousal measures were linked to ADHD symptoms independent of ODD/CD.

## 1 Introduction

Attention-deficit/hyperactivity disorder (ADHD) is a common neurodevelopmental disorder with postulated links to hypo-arousal and arousal dysregulation. The state regulation and cognitive-energetic accounts suggested that a sub-optimal arousal state in ADHD may lead to inconsistent cognitive performance, reflected for example by within-subject fluctuations in reaction time (Van Der Meere, 2005, Sergeant, 2005). Recent initial findings from our research group have suggested that hypo-arousal, while observed during performance on a low-demand reaction time task, is not stable in individuals with ADHD but may be normalized during more stimulating tasks (James et al., 2016). More research is needed to understand the physiological underpinnings of ADHD by exploring whether ADHD case-control differences are context-dependent or stable across time, and whether these differences are specific to ADHD or can be explained by other related behaviors.

Skin conductance provides an objective and reliable index of arousal in the peripheral nervous system (Boucsein, 2012). SC is a measure of electrodermal activity, which is stimulated by the autonomic sympathetic nervous system, a system involved in regulating arousal and alertness (Boucsein, 2012, Critchley, 2002). In this study, we use the term ‘arousal’ to describe changes in electrodermal activity. Skin conductance level represents the tonic level of arousal (average level) and non-specific fluctuations represent a phasic (transient) change in arousal. Increased skin conductance level indexes an increase in peripheral arousal (Boucsein, 2012), whereas increased non-specific fluctuations indicate more variability in arousal.

Several studies have reported attenuated skin conductance level in children with ADHD, compared to controls, indicating hypo-arousal during resting-state (eyes open and eyes closed) and task conditions (Barry et al., 2012, Conzelmann et al., 2014, Dupuy et al., 2014, Iaboni et al., 1997, Lazzaro et al., 1999, Mangeot et al., 2001, Mangina et al., 2000, O'Connell et al., 2004). Research is more limited in adults, where study findings across resting-state and task conditions are inconclusive in terms of hypo-arousal in ADHD (Mayer et al., 2016, Hermens et al., 2004). Mixed findings, mainly from studies on younger children and adolescents with ADHD, have emerged also for non-specific fluctuations. While several studies on children and adolescents with ADHD reported significantly fewer non-specific fluctuations in their electrodermal activity during resting conditions than controls (Beauchaine et al., 2001, Crowell et al., 2006, Satterfield and Dawson, 1971), other studies in children have not replicated these findings (Beauchaine et al., 2015) and one study even found the opposite direction of effects in a resting condition while participants listened to 40-decibel white noise (Pliszka et al., 1993). Further research is needed using large samples, across testing conditions, to clarify these inconsistencies in the literature.

Our understanding is also limited regarding the specific aspects of ADHD that arousal measures tap into. Only one study, which consisted of girls with and without ADHD, has explored the relationship between skin conductance level and the two ADHD symptoms domains separately, reporting that lower skin conductance level was strongly correlated with higher inattentive symptoms (*r* = −0.45) and weakly-to-moderately correlated with hyperactive-impulsive (*r* = −0.23) symptoms, in individuals with and without ADHD (Dupuy et al., 2014). No study to our knowledge has explored this with non-specific fluctuations, and the relationship between skin conductance level and non-specific fluctuations remains poorly understood. Studies in children have reported that non-specific fluctuations correlate positively with average skin conductance level (Burch and Greiner, 1960, Silverman et al., 1959); yet neuroimaging and electrophysiological studies suggest that non-specific fluctuations and skin conductance level index different underlying processes (Lazzaro et al., 1999, Nagai et al., 2004).

In a recent investigation with a large sample of adolescents and young adults, we found that individuals with ADHD displayed autonomic under-arousal during a baseline (slower, non-rewarded) task condition of a four-choice reaction time called the Fast Task, but this was normalized in a more stimulating fast-incentive condition (James et al., 2016). These findings support an arousal dysregulation account of ADHD rather than suggesting that individuals with ADHD display stable hypo-arousal. Further support for this view comes from a study that investigated autonomic arousal measures in participants during a sustained attention to response task before and after taking part in either self-alert training, where participants learned to modulate their arousal levels, or placebo training (O'Connell et al., 2008). Results showed that both ADHD and control participants had increased specific skin conductance responses, indicating increased phasic arousal, after the alertness training. Another study in a healthy population sample found increased skin conductance level during a continuous performance task compared to baseline, a difference defined as ‘activation’, which further suggests context-dependent effects of autonomic arousal (Vaez Mousavi et al., 2009). We now investigate context effects in ADHD further by studying tonic (skin conductance level) and phasic (non-specific fluctuations) autonomic arousal across a longer experimental assessment, to improve our understanding of the stability of autonomic arousal profiles in ADHD.

In this study we firstly aim to (1) extend initial findings from James et al. (2016) and investigate if ADHD case-control differences in both tonic arousal, indexed by skin conductance level, and phasic arousal, indexed by non-specific fluctuations, vary across a long testing session consisting of a combination of resting-state and task conditions (Resting-state time 1, Continuous Performance Task (CPT-OX), Fast Task: Baseline and Fast-Incentive condition, Resting-state time 2) commonly used in ADHD research, in a large sample of adolescents and young adults. We then more specifically aim to (2) examine if ADHD case-control differences in autonomic arousal measures vary across time from Resting-state time 1 to time 2. These findings may provide insight on whether low levels and fluctuating arousal in ADHD reflect context-specific states or stable traits. This in turn would be relevant both for our understanding of the nature of biological underpinnings of ADHD and potentially for treatment, as modifiable markers of ADHD may be suitable targets for interventions, such as biofeedback paradigms. Thirdly, to further understand which aspects of ADHD specifically tap into tonic and phasic arousal, we aim to investigate (3) how arousal measures are associated with each of the ADHD symptom domains of inattention and hyperactivity/impulsivity.

In all analyses, we also aim to investigate if associations between ADHD and arousal are independent of oppositional defiant disorder and conduct disorder (ODD/CD) symptoms, which frequently co-occur with ADHD and have previously been associated with lower skin conductance level and skin conductance responses (Delamater and Lahey, 1983, Fung et al., 2005, Posthumus et al., 2009). One small study of males found that among individuals with ADHD, those with and without comorbid CD showed similar profiles of fewer non-specific fluctuations during a baseline resting condition compared to controls (Beauchaine et al., 2001); however, more powerful studies including both males and females are needed to determine the specificity of arousal profiles in ADHD.

## 2 Materials and methods

### 2.1 Participants

The original sample (before quality control and exclusions) consisted of 275 participants, followed-up on average 5.8 years (SD = 1.1) after initial assessments. At follow-up, participants were on average 18.0 years of age (age range: 11.1–25.9). 108 participants had a diagnosis of DSM-IV combined type ADHD in childhood (9 sibling pairs, 90 singletons) and 167 were controls (74 sibling pairs, 19 singletons).

Participants with ADHD were initially recruited from ADHD clinics in south-east England (Kuntsi et al., 2010). Diagnosis of DSM-IV combined type ADHD was established using the Parental Account of Childhood symptoms (PACS), a semi-structured interview with high inter-rater reliability (Chen et al., 2008). The control group was initially recruited from schools in the UK, aiming for an age and gender match with the clinical sample. For this analysis, there were no differences in age and sex between the control and clinical sample (Table 1) and re-running the main analyses controlling for age and sex did not change the pattern of results. All participants were aged between 6 and 17 at initial assessment. Exclusion criteria were: IQ < 70, autism, epilepsy, brain disorders and any genetic or medical disorder associated with externalizing behaviors that might mimic ADHD. At follow up, eight controls met DSM-IV ADHD criteria based on parent-ratings (*n* = 6) on the Barkley Informant Rating Scale (Barkley and Murphy, 2006); these participants were excluded from analyses. The investigation was carried out in accordance with the latest version of the Declaration of Helsinki.

**Table 1:** Descriptives and pair-wise comparisons between Groups (ADHD, control) in each condition on skin conductance measures.

		ADHD (71)	Control (140)	*t/χ²*	*p*	Cohen's *d*	Cohen's *d* Cov: ODD/CD
**Male sex, *n* (%)**		59 (83%)	107 (76%)	0.63	0.43	0.17	
**IQ, *M* (*SD*)**		95.38 (14.97)	110.08 (12.69)	7.47	0.001	−1.03	
**Age, *M* (*SD*)**		17.70 (2.83)	17.75 (2.28)	0.20	0.66	0.02	
**Resting-state time 1**	*SCL*	2.88 (2.07)	3.29 (2.26)	−1.43	0.31	−0.20	−0.18
	*NSF/s*	.07 (0.05)	.06 (0.06)	0.33	0.74	0.05	0.01
**CPT-OX**	*SCL*	3.72 (2.16)	4.24 (2.68)	−0.96	0.32	−0.13	−0.09
	*NSF/s*	.23 (0.13)	.23 (0.13)	0.02	0.98	0.01	0.01
**Fast Task: Baseline**	*SCL*	2.96 (2.05)	4.16 (1.91)	−3.44	<0.01	−0.49*	−0.56*
	*NSF/s*	.10 (0.04)	.08 (0.04)	2.06	0.04	0.33*	0.27
**Fast Task: Fast-Incentive**	*SCL*	4.84 (2.03)	5.45 (3.06)	−1.47	0.15	−0.26	−0.23
	*NSF/s*	.11 (0.04)	.11 (0.05)	−0.66	0.51	0.12	0.08
**Resting-state time 2**	*SCL*	4.28 (2.23)	4.73 (2.77)	−0.41	0.69	−0.06	−0.01
	*NSF/s*	.09 (0.06)	.07 (0.06)	2.14	0.03	0.32*	0.30*

*Note.* Data on SCL from the Fast Task have already been presented (James et al., 2016), but for ease of comparison, results specific to this analysis have been replicated here with the additional results across other task conditions.

⁎ *p* < 0.05. Cov: Covariate included in models. CPT-OX: Continuous performance task. SCL: Skin conductance level. NSF/s: Non-specific fluctuations per second.

Skin conductance data were available for 221 participants (mean age: 17.7 years, age range: 11.9–23.3), out of our original sample of 256, as skin conductance data collection equipment did not arrive until after the initial participants had been assessed. We additionally excluded participants within each testing condition (percentage of excluded individuals across conditions: 10%−19%) who experienced skin conductance equipment failure or extreme drowsiness. The final sample consisted of 71 ADHD probands and 140 controls. The ADHD and control groups did not differ in age (*t* = 0.20, *p* = 0.66), gender (χ2 = 0.63, *p* < 0.43), but did differ on IQ scores (*t* = −7.47, *p* < 0.001; Table 1).

### 2.2 Materials and procedure

Participants with childhood ADHD were classified as having ADHD if they met DSM-IV criteria for ADHD at follow-up. If they scored a ‘yes’ on ≥ 6 items in either the inattention or hyperactivity-impulsivity domains of the Diagnostic Interview for ADHD in adults (DIVA; Kooij and Francken, 2007) and if they scored ≥ 2 on two or more areas of impairments from the Barkley's functional impairment scale (BFIS; Barkley and Murphy, 2006), they were classified as ADHD persisters at follow-up. Out of the 108 participants with childhood ADHD, 23 were classified as ADHD ‘remitters’ at follow-up and were not included in this study.

#### 2.2.1 Conners’ parent rating scale – revised (L)

is a questionnaire measure used to assess internalizing and externalizing behavior from children and adolescents based on parent ratings. The scale includes 18 statements that measure DSM-IV inattentive and hyperactive-impulsive ADHD symptoms (Conners, 1997). Each statement is rated on a three-point scale, by parents, and the highest possible score is 54. These subscales were used in the correlation analyses as they were assessed in both ADHD cases and controls.

#### 2.2.2 The development and well-being assessment (DAWBA; Goodman et al., 2000)

is a structured interview administered by lay interviewers. The K-section of the DAWBA questionnaire, which measures ‘behaviors which sometimes gets children into trouble’, was administered to participants. These items reflect current symptoms, closely related to DSM-IV diagnostic criteria of ODD and CD (Heiervang et al., 2007, Goodman et al., 2000).

#### 2.2.3 IQ

The vocabulary and block design subtests of the Wechsler Abbreviated Scale of Intelligence (WASI) were administered to derive an IQ estimate (Wechsler, 1999). The WASI subtests have shown strong correlation with full-scale IQ (*r* = 0.83–0.88; Wechsler, 1991) and other measures of intelligence (*r* = 0.66–0.89; Canivez et al., 2009, Hays et al., 2002).

#### 2.2.4 Resting-state with eyes open

Participants were asked to keep as still as possible while resting in a chair with their eyes open before and after the cognitive assessments. They were encouraged to find a spot on the wall in front of them where they could fixate their gaze. The resting-state sessions each lasted for 3 min.

#### 2.2.5 The Fast Task; baseline and fast-incentive condition (Andreou et al., 2007)

The baseline condition of the Fast Task consists of 72 trials, which followed a standard warned four-choice RT task. Four empty circles (warning signals, arranged horizontally) first appeared for 8 s, after which one of them (the target) was colored in. Participants were asked to press the response key that corresponded to the target position. Following a response, the stimuli disappeared and a fixed inter-trial interval of 2.5 s followed. Speed and accuracy were emphasized equally. If participants did not respond within 10 s, the trial terminated. A comparison condition of 80 trials with a fast event rate (fore-period of 1 s) and incentives followed the baseline condition. The fast-incentive condition is always administered after the baseline condition. Owing to the longer fore-period in the slow condition, the two conditions were not matched on task length, but they were matched on the number of trials.

#### 2.2.6 The cued flanker Continuous Performance Task (CPT-OX)

This CPT-OX (Doehnert et al., 2008, Valko et al., 2009) includes rare cued Go and NoGo conditions embedded in a vigilance task with frequent distractors to assess attentional and inhibitory processes. The test originates from the AX Continuous Performance Task (Rosvold, 1956) and stimuli were flanked by adjacent incompatible distractors, similar to the classic flanker paradigm (Eriksen and Hoffman, 1973), to increase task difficulty (McLoughlin et al., 2010). The test consists of 400 letters presented in a pseudo-randomized order for 150 ms every 1.65 s. The cue letter O occurred with 20% probability (80 Cue stimuli), signaled a Go-NoGo task, and induced response preparation. Participants pressed a mouse button as fast as possible every time the cue was followed directly by the letter X (O-X) target sequence, 10% probability, 40 Go stimuli] but had to withhold responses to O-not-X sequences (NoGo trials, also 10%, 40 NoGo stimuli).

### 2.3 Procedure

Participants were re-contacted by telephone and scheduled for a follow-up clinical interview and cognitive assessments at our research Centre while electrodermal and electroencephalogram (EEG) measures were recorded. Before the cognitive assessments, participants were asked to remain still and rest with their eyes open while fixating at a point in front of them for 3 min. They then performed the CPT-OX for 11 min, followed by the Fast Task baseline condition for 13 min, and were asked to rest again with their eyes open for 3 min at the end of the testing session. A 48 h ADHD medication-free period was required and the participants were also asked to abstain from caffeine, smoking, and alcohol on the day of testing.

### 2.4 Skin conductance

Skin conductance response was recorded using PSYCHLAB SC5 24 bit equipment system, which has an absolute accuracy of ±0.1 microsiemens. The SC5 is connected to a computer that runs the PSYCHLAB software where the data can be monitored and recorded in real time and parameters can be set. Skin conductance was measured by attaching a pair of 8 mm diameter silver-silver chloride electrodes on the palm of participants’ non-dominant hand (thenar eminence and hypothenar eminence) at the beginning of the cognitive test battery. An electrode paste, formulated with 0.5% saline in a neutral lotion/cream style base (provided by PSYCHLAB), was used to establish a stable electrical skin conductance signal. The SC5 is DC coupled (infinite time constant), and a constant imperceptible voltage (0.5 V) was applied. SC5 automatically calibrates itself when switched on and then runs at a fixed internal sample rate of 80 Hz and an additionally 10 Hz filter is applied to response signal to prevent aliasing.

Skin conductance variables were calculated using a in-house system that is based on a skin conductance sigmoid-exponential model that allows the tonic measure of skin conductance level to be disentangled from phasic skin conductance fluctuations and allows the decomposition of overlapping skin conductance fluctuations (Lim et al., 1997). The statistical model was applied to each task condition. Each participant's data were inspected visually by a researcher to confirm that the data were scored properly using the statistical model. Each non-specific fluctuation reflects a rise in skin conductance level for at least 500 milliseconds followed by at least 300 milliseconds of non-rising skin conductance, and the minimum amplitude of the non-specific fluctuations was set to 0.02 microsiemens. The number of non-specific fluctuations per second was used as the final measure to control for minor individual differences in recording lengths. Mean skin conductance level and non-specific fluctuations per second were calculated for each participant in each testing condition. We examined average measures of skin conductance level and non-specific fluctuations across task performance in the CPT-OX and Fast Task, and did not exclude event-locked skin conductance variables, as event codes during the CPT-OX were not retrievable in these data.

### 2.5 Data analysis

We ran regression models to investigate ADHD case-control group differences in skin conductance level and non-specific fluctuation measures within each testing condition (Resting-state time 1, CPT-OX, Fast Task: Baseline and Fast-Incentive condition, Resting-state time 2). Relatedness between sibling pairs was controlled for by using the ‘robust cluster’ command in STATA (StataCorp, College Station, TX). Random-intercept linear models were used to test the main and interaction effects of group (ADHD cases, controls) and time (Resting-state time 1, Resting-state time 2) on skin conductance level and non-specific fluctuations, to examine the change in autonomic arousal in ADHD and control groups over time. Random-intercept models control for clustered data, due to relatedness between siblings, and handle missing data using the maximum likelihood method, which in turn reduces the loss in power from missing data points. We re-ran all analyses controlling for ODD/CD symptoms (using DAWBA) to examine if any identified ADHD case-control differences could be explained by co-occurring ODD/CD symptoms. We did not control for internalizing symptomology based on prior work with this sample that showed that controlling for internalizing symptoms did not influence the results (James et al., 2016).

We ran linear regression models to investigate the associations of skin conductance measures with inattentive and hyperactive-impulsive symptoms, respectively, and added an interaction term (skin conductance*ADHD group) to investigate if the strength of the associations were different in the ADHD and control groups. We tested these associations only in conditions that showed sensitivity to ADHD as indicated by a significant case-control difference in skin conductance measures. We re-ran all analyses controlling for ODD/CD symptoms.

We re-ran the main analyses on ADHD case-control group comparisons with IQ added as a covariate to examine its potential effects. We further ran sensitivity analyses testing age and gender as covariates in the main analyses in line with previous analyses in the same sample (Kitsune et al., 2015). The effects of potential longer-term use of medication on skin conductance measures were examined by running skin conductance comparison tests between unmedicated and medicated participants with ADHD.

## 3 Results

### 3.1 ADHD case-control differences in arousal across testing sessions

**Fig. 1:**
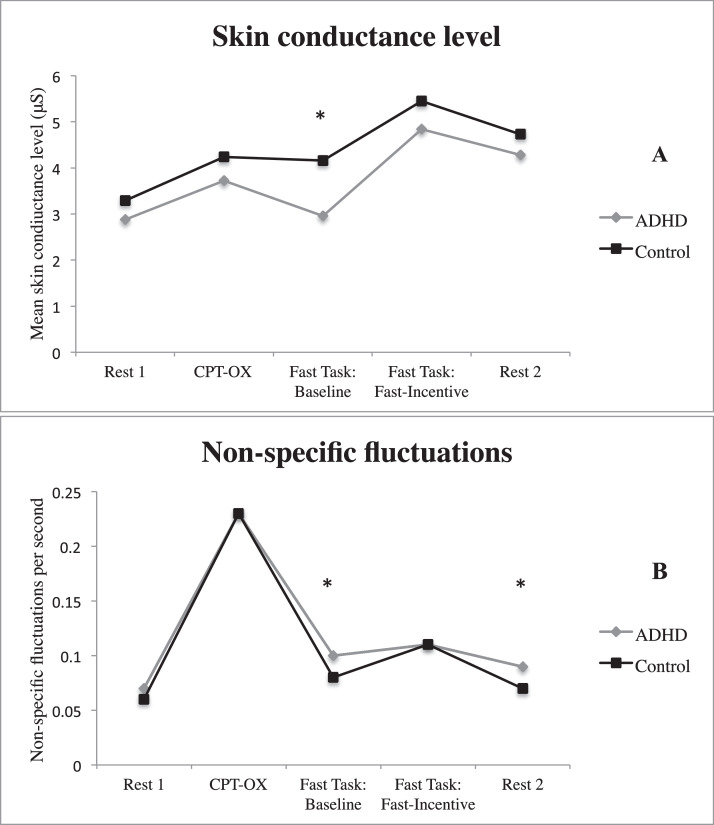
Mean skin conductance level (A) and non-specific fluctuations (B) for ADHD and control groups in each testing condition. *Note.* Data on SCL from the Fast Task have already been presented (James et al., 2016), but for ease of comparison, results specific to this analysis have been replicated here with the additional results across other task conditions. * *p*-value < 0.05 for comparison between ADHD-control groups. CPT-OX: continuous performance task.

Pairwise comparisons revealed no significant differences (*p* > 0.05) between the ADHD and control groups in non-specific fluctuations or skin conductance level during the two initial testing conditions (Resting-state time 1 and CPT-OX task performance) and the Fast-Incentive condition of the Fast Task (Fig. 1). During performance on the Baseline condition of the Fast Task and Resting-state time 2, individuals with ADHD showed significantly more non-specific fluctuations than the control group. There was no significant group difference in skin conductance level during Resting-state time 2, in contrast to the significantly lower skin conductance level found during the Baseline condition of the Fast Task, as reported James et al. (2016; Fig. 1, Table 1).

Individuals with ADHD had a significantly higher level of ODD/CD symptoms (*M* = 4.03, *SD* = 2.63) than individuals in the control group (*M* = 1.61, *SD* = 1.90; *t*(210) = 6.87, *p* < 0.001). After controlling for ODD/CD symptoms in the models, skin conductance level findings of case-control differences did not change but non-specific fluctuation findings during the Baseline condition of the Fast Task changed slightly, in regards of the effect size (Cohen's *d*: from 0.33 to 0.27) and *p*-value (from 0.04 to 0.07; Table 1), although an overlap in 95% confidence intervals of coefficients indicated that the change in results was not significant (95% CI [0.01, 0.70] to [−0.17, 0.17]).

### 3.2 ADHD case-control differences in arousal across time

The random-intercept models revealed significant main effects of time (Resting-state time 1 vs 2) on non-specific fluctuations and skin conductance level (Table 2), showing that non-specific fluctuations and skin conductance level significantly increased over time. We found no significant main effect of group (ADHD vs control) or group-by-time interaction effects on non-specific fluctuations or skin conductance level (Table 2). When ODD/CD symptoms were controlled for, the group-by-time interaction effect became significant for non-specific fluctuations (*z* = 2.00, *p* = 0.045). Post-hoc analyses when controlling for ODD/CD symptoms revealed significant increases in non-specific fluctuations from resting-state time 1 to time 2 in the ADHD group (*t* = 3.32, *p* = 0.002), but not in the control group (*t* = 0.63, *p* = 0.53).

**Table 2:** Main effects of Group (ADHD vs Control), Time (Resting-state time 1 vs 2) and interaction effects of Group-by-Time on skin conductance measures.

	Skin conductance level		Non-specific fluctuations	
	*z*	*p*	*z*	*p*
**Group**	−0.94	0.35	1.61	0.11
**Time**	8.91	0.001*	2.71	0.01*
Group*Time	0.38	0.71	1.53	0.12*

⁎ *p* < 0.05 after controlling for ODD/CD.

As the group-by-time interaction effect on non-specific fluctuations emerged as significant after controlling for ODD/CD symptoms, we decided to run regression analyses to explore the associations between ODD/CD symptoms and skin conductance measures, within each group (ADHD, control) and each condition. We found no significant associations between ODD/CD symptoms and skin conductance in any of the groups or conditions (Table A.1).

### 3.3 Linear associations between arousal measures and each ADHD symptom domain

Linear regression models revealed that non-specific fluctuations recorded during the Fast Task was significantly and positively associated with hyperactive-impulsive symptoms in the full sample, and non-specific fluctuations during Resting-state time 2 was significantly and positively associated with both inattentive and hyperactive-impulsive symptoms (Table 3). Skin conductance level recorded during the Fast Task was significantly and negatively associated with both symptom domains. While non-specific fluctuations during the Fast Task was not significantly associated with inattentive symptoms in the full sample, the non-specific fluctuations-by-group interaction was significant, revealing that the association between non-specific fluctuations and inattentive symptoms was significant in the ADHD group (*Beta* = 0.13, *p* = 0.01), but not in the control group (*Beta* = −0.01, *p* = 0.77) (see scatterplots in Figure A.1). No other interaction terms (skin conductance*group) were significant (Table 3). When we controlled for ODD/CD symptoms in the regression models, the pattern of results did not change with regard to significance level (Table 3).

**Table 3:** Main associations between skin conductance level and non-specific fluctuations with ADHD symptom domains and skin conductance-by-group (ADHD, control) effects on ADHD symptom domains.

	Fast Task								Resting-state time 2			
	Non-specific fluctuations				Skin conductance level				Non-specific fluctuations			
	Main association		Interaction (NSF*group)		Main association		Interaction (SCL*group)		Main association		Interaction (NSF*group)	
	*B*	*P*	*B*	*p*	*B*	*P*	*B*	*P*	*B*	*p*	*B*	*p*
**Hyperactivity-Impulsivity**	**0.20**	<0.01*	−0.06	0.66	**−0.26**	<0.01*	−0.15	0.19	**0.18**	0.02*	−0.01	0.98
**Inattention**	0.10	0.19	−**0.12**	0.04*	**−0.26**	<0.01*	−0.10	0.10	**0.18**	0.02*	−0.02	0.77

Note: Associations between ADHD symptoms and arousal measures were only tested during the conditions that revealed significant case-control differences in arousal.

⁎ *p* < 0.05 after controlling for ODD/CD.

### 3.4 Sensitivity analyses

We re-ran the main pairwise comparisons of groups (ADHD vs Control) with IQ added as a covariate (Table A.2). We found that the pattern of results remained the same for non-specific fluctuations but for skin conductance level the ADHD case-control difference during the Baseline condition of the Fast Task was no longer significant and the effect size (Cohen's d) changed from −0.49 to −0.09 (Table 1), although 95% CI's showed an overlap before and after controlling for IQ (95% CI [−0.95, −0.28] to [−0.58, 0.15]).

Due to the changes in results after controlling for IQ in the skin conductance level analyses (Table A.2), we ran additional sensitivity analyses to investigate the associations between IQ and skin conductance measures across conditions (see Table A.3 and A.5).

We further re-ran the main pairwise comparisons of groups (ADHD vs Control) with age and gender added as covariates and found that the pattern of findings remained the same in terms of significance of findings (Table A.4). Furthermore, analyses were re-run excluding siblings, and the pattern of findings remained consistent without these cases of non-independence. We also ran skin conductance comparison tests between unmedicated and medicated participants with ADHD to investigate the long-term effects of medication. Short-term effects of medication were controlled for, as participants were asked to have a 48 h medication-free period before testing. There were no significant differences in skin conductance level (*t*(34) = 0.68, *p* = 0.50) or non-specific fluctuations (*t*(35) = −0.48, *p* = 0.64) between unmedicated and medicated participants.

As we found significant ADHD case-control differences in skin conductance during the 13 min long Baseline condition of the Fast Task but not during the 11 min long CPT-OX, we aimed to explore whether the significant differences during the Baseline condition of the Fast Task emerged because of the longer testing session, rather than the order or nature of the task. James et al. (2016) previously demonstrated that the significant ADHD case-control difference in skin conductance level during the Baseline condition of the Fast Task was consistent across time, as significant differences were found in each 4 min long snippet of the task. Here, we extracted non-specific fluctuation data during the first 11 min of the Baseline condition of the Fast Task to match the CPT-OX on task length, and re-ran the ADHD case-control comparisons. We found that the ADHD case-control difference in non-specific fluctuations during the Baseline condition of the Fast Task was reduced to trend level when using the shorter 11 min time period (*Beta* = 0.03, *p* = 0.068).

### 3.5 Follow-up tests of robustness

A false discovery rate-controlling analysis (Benjamini and Hochberg, 1995, Benjamini and Hochberg, 2000) was conducted to test the robustness of effects in relation to the many tests that were run and to control for the expected proportion of false discoveries (Type I Error). All observed p-values were ordered sequentially from low (*p*_1_) to high (*p*_m_), where m represents the total number of *p*-values from the main analyses (*m* = 28). The largest k was then identified such that *p*_k_ < 0.05 * k/m and the adjusted alpha level of 0.05 * k/m was 0.011. All of the reported significant effects relating to skin conductance level had *p*-values below the adjusted alpha. However, several of the reported effects in relation to skin conductance fluctuations had *p*-values above the adjusted alpha level: the ADHD case-control difference in non-specific fluctuations in both the Baseline condition of the Fast Task (*p* = 0.04) and the Resting-state time 2 (*p* = 0.03), the Group*Time interaction effect after controlling for ODD/CD (*p* = 0.045) and the associations between ADHD symptoms and non-specific fluctuations during Resting-state time 2 (*p* = 0.02). These latter results should therefore be interpreted with caution and may represent trends rather than significant effects.

## 4 Discussion

In this large physiological study of 211 adolescents and young adults, we found that autonomic arousal profiles of individuals with ADHD varied across testing conditions. First of all, ADHD case-control differences in tonic arousal, indexed by skin conductance level, only emerged during a slow and low-demanding cognitive task. Case-control differences in phasic arousal, indexed by non-specific fluctuations, emerged towards the end of the assessments, during the low-demanding cognitive task and the final resting-state condition (time 2). Further analyses showed that case-control differences in phasic arousal, but not tonic arousal, emerged over time from resting-state time 1 to time 2, once ODD/CD symptoms were controlled for. It is, however, important to note that after correcting for multiple testing, the findings on phasic arousal emerged as trends rather than as significant effects. Lastly, both ADHD symptom domains were significantly associated with lower levels of tonic arousal and more fluctuating arousal, independently of ODD/CD symptoms. Overall, our findings suggest that individuals with ADHD experience difficulties regulating their arousal rather than being constantly under-aroused. Inconsistent findings in the literature on autonomic arousal in ADHD might be explained by differences in experimental designs and tasks.

Extending the initial report from James et al. (2016), we now show that tonic autonomic arousal, measured by skin conductance level, did not remain significantly lower in individuals with ADHD compared to controls beyond the slow, baseline condition of the Fast Task; during resting-state conditions or performance of the high-demanding CPT-OX or the Fast-Incentive condition. These findings suggest that lower arousal levels in individuals with ADHD may be especially salient during slow and low-demanding tasks compared to faster-paced and more demanding tasks such as the high-demanding CPT-OX or the fast-incentive condition of the Fast Task (as also demonstrated in James et al., 2016). We were unable to separate fatigue effects from effects of cognitive demand, as the tasks were not counterbalanced in this experiment. However, as the lower arousal level in the ADHD group was found in the Baseline condition of the Fast Task but not the Fast-Incentive condition, which was performed directly after the Baseline condition, the group difference in arousal level is likely not due to fatigue effects. Our analyses further revealed that lower tonic arousal during the Baseline condition of the Fast Task, where case-control differences were identified, was associated with a higher level of inattentive and hyperactive-impulsive symptoms, supporting initial findings from a study only in girls (Dupuy et al., 2014). Our finding suggests that individuals with ADHD may experience difficulties in regulating their arousal levels rather than experience constant hypo-arousal, which implies that arousal is malleable in individuals with ADHD and may therefore be suitable as a potential treatment target. Our findings further suggest that inconsistencies in the literature (Hermens et al., 2004, Mayer et al., 2016) may be explained by the different experimental paradigms used across studies.

We further found that individuals with ADHD displayed more fluctuating arousal, indexed as a higher number of non-specific fluctuations per second, compared to controls, during the baseline condition of the Fast Task and Resting-state time 2 only. These effects were however not significant when false discovery rate-controlling analysis was applied and may therefore be interpreted as trends. These findings suggest that arousal variability in ADHD, similarly to under-arousal, may become more salient during slower and low-demanding tasks, but also towards the end of assessment, over time. This is further supported by our sensitivity analysis showing that the ADHD case-control difference in non-specific fluctuations during the 13 min long Fast Task was no longer significant when we shortened the task to closer match the lengths of the CPT-OX and Fast-Incentive condition of the Fast Task. These findings indicate that more fluctuations in ADHD may become especially salient over time, possibly in combination with the low-demanding task.

Our results further showed that the fluctuations in and level of arousal increased over time, from resting-state time 1 to time 2. When we tested the group-by-time interaction on fluctuations, the effect emerged as significant (trend after false discovery rate-controlling analysis) once ODD/CD symptoms were controlled for and post-hoc analyses revealed that the fluctuations in arousal increased over time only in the ADHD group. These findings further suggest that fluctuating arousal profiles in ADHD, relative to controls, become more salient over time; an effect that is enhanced by controlling for other co-occurring externalizing behaviors.

Non-specific fluctuations were associated with both inattentive and hyperactive-impulsive symptoms, across groups, in the testing conditions that showed case-control differences, with the only exception of the Fast Task (Baseline condition) where non-specific fluctuations and inattention were only significantly associated in the ADHD group. Overall, we found that individuals with ADHD did not show stable abnormalities in fluctuating arousal, similarly to under-arousal, which may in turn explain highly inconsistent findings in the literature where different experimental designs have been used. The direction of effects is in line with findings from one previous study that showed a trend of more non-specific fluctuations in children with ADHD compared to controls (Pliszka et al., 1993), but is inconsistent with other studies of children and adolescents which have found opposite effects of less frequent non-specific fluctuations in individuals with ADHD (Lazzaro et al., 1999, Satterfield and Dawson, 1971). Further research across different experimental conditions and age groups, is therefore needed to clarify the discrepancy in findings.

This is the first larger study, to our knowledge, to investigate the specificity of both phasic and tonic arousal profiles in young adults with ADHD by controlling for ODD/CD symptoms in analyses. ODD/CD symptoms did not account for our findings on atypical tonic arousal profiles in ADHD, which is in line with previous research (Beauchaine et al., 2001, Van Lang et al., 2007). For phasic arousal, ODD/CD symptoms did not account for the associations with ADHD, however, the group-by-time effect emerged as significant (trend after false-discovery rate-controlling analysis) after controlling for ODD/CD symptoms. This suggests that controlling for ODD/CD symptom enhances the relationship between phasic arousal and ADHD over time, but it is not clear from these results how ODD/CD symptoms relate to the other variables, as they (a) do not account for the linear associations between non-specific fluctuations non-specific fluctuations and ADHD symptom domains (Table 3) and (b) are not significantly associated with non-specific fluctuations (Table A.1). While previous research has suggested that individuals with antisocial/conduct problems have smaller amplitude of specific skin conductance responses (Delamater and Lahey, 1983), less is known of non-specific fluctuations. Further studies are needed to clarify the complex relationship between ODD/CD, non-specific fluctuations and ADHD, to determine the specificity of fluctuating arousal profiles in ADHD.

A limitation of this study is that we used measures of non-specific fluctuations averaged across each of the CPT-OX and Fast Task conditions, as we were unable to retrieve event codes. This means that we could not tease apart skin conductance fluctuations during stimuli presentation and response execution from skin conductance fluctuations during no task events. It would have been interesting to study both event-specific and non-specific fluctuations separately to explore how they each are implicated in ADHD, however, given that very few studies have investigated arousal variability in ADHD, we believe it is still meaningful to study average fluctuations in our rich dataset that spans across a long testing session. Another issue to highlight is that we had limited power to account for all relevant covariates in one model, however, we controlled for these variables in separate models, which allowed us to gain an understanding of their unique impact on the results. Finally, we found that the ADHD case-control difference in tonic arousal was largely accounted for by IQ, suggesting that it is important to take IQ into consideration in future studies that investigate arousal levels in ADHD.

### 4.1 Correction for multiple testing

We did not correct for multiple testing in our initial main analyses because of the exploratory nature of the investigations into context effects on ADHD case-control differences in skin conductance level and non-specific fluctuations. In our study, analyses were restricted to skin conductance measures that were expected to be sensitive to impairments in ADHD, in order to reduce the chance for false negative findings from multiple testing. Further, in the interpretation of results, the emphasis was places on both effect sizes and significance to provide a complete picture of the full impact of results. We did however run sensitivity tests where we applied a false-discovery rate-controlling analysis. While all analyses on tonic arousal remained significant, several of the analyses on phasic arousal emerged as trends rather as significant, with *p*-values above the adjusted alpha level. The significant association between phasic arousal during the baseline condition of the Fast Task and ADHD symptoms, however, remained as significant after the multiple testing correction. Future replication of our results is important to validate findings before drawing firm conclusions and applying implications of findings in practice.

## 5 Conclusions

We found that adolescents and young adults with ADHD displayed lower levels of and more fluctuating autonomic arousal under certain experimental conditions. ADHD case-control differences in tonic arousal emerged only during a slow, low-demanding cognitive task. A case-control difference in phasic arousal was also observed during the low-demanding task and also towards the end of the assessment. We further found that tonic and phasic arousal were associated with both inattentive and hyperactive-impulsive symptoms, independently of ODD/CD symptoms. Our findings suggest that both tonic and phasic autonomic arousal profiles in ADHD are context-specific rather than representing stable impairments. Our findings also highlight how inconsistent findings in the ADHD literature on arousal may be explained by differences in experimental paradigms used across studies.

## Declaration of interest

Professor Banaschewski has served as adviser or consultant for Bristol Myers-Squibb, Develco Pharma, Lilly, Medice, Novartis, Shire, and Vifor Pharma; he has received conference attendance support and conference support or speakers honoraria from Janssen McNeil, Lilly, Medice, Novartis, and Shire and has been involved in clinical trials conducted by Lilly and Shire. Professor Asherson is supported by the National Institute for Health Research (NIHR) Biomedical Research Centre at South London and Maudsley NHS Foundation Trust and King's College London, and by an NIHR senior Investigator Award (NF-SI-0616-10040). Professor Asherson has acted in an advisory role for Shire, Janssen-Cilag, Eli-Lillyand Flynn Pharma. He has received education or research grants from Shire, Janssen-Cilagand Eli-Lilly. He has given talks at educational events sponsored by the above companies. Professor Daniel Brandeis serves as an unpaid scientific advisor for an EU funded neurofeedbackstudy. Professor Jonna Kuntsi has given talks at educational events sponsored by Medice; all funds are received by King's College London and used for studies of ADHD. Dr Ebba Du Rietz and Dr Sarah Naomi James report no biomedical financial interests or potential conflicts of interest.

## Appendix Supplementary materials

Image, application 1
