# Supplementary material for: Autonomic arousal profiles in adolescents and young adults with ADHD as a function of recording context
Source: Psychiatry Res. 2019 May;275:212–20. doi: 10.1016/j.psychres.2019.03.039 (PMC6525183; doi:10.1016/j.psychres.2019.03.039)
Supplement: Supplementary file 1 [file mmc1.docx]

**Supplementary material**

|  | **NSFs** | | | | **SCL** | | | |
| --- | --- | --- | --- | --- | --- | --- | --- | --- |
|  | ADHD | | Controls | | ADHD | | Controls | |
| **ODD/CD** | *Beta* | *p* | *Beta* | *p* | *Beta* | *p* | *Beta* | *p* |
| **Rest1** | 0.01 | 0.92 | 0.04 | 0.50 | -0.15 | 0.41 | 0.03 | 0.63 |
| **CPT-OX** | -0.04 | 0.79 | 0.02 | 0.77 | -0.15 | 0.33 | 0.05 | 0.48 |
| **Fast Task: Baseline** | 0.05 | 0.40 | -0.02 | 0.78 | 0.11 | 0.44 | -0.01 | 0.98 |
| **Fast Task: Fast-Incentive** | -0.11 | 0.52 | 0.02 | 0.79 | -0.14 | 0.51 | 0.05 | 0.37 |
| **Rest 2** | -0.04 | 0.77 | 0.09 | 0.13 | -0.03 | 0.85 | -0.02 | 0.84 |

**Table A.1: Associations between skin conductance measures and oppositional defiant disorder/conduct disorder symptoms within each (ADHD and control) group.**

*ODD/CD: Oppositional defiant disorder/conduct disorder symptoms. CPT-OX: Continuous performance task. SCL: Skin conductance level. NSFs: non-specific fluctuations per second.*

** p-value < 0.05 after controlling for ADHD symptoms*

**Table A.2: Pair-wise tests between groups (ADHD and control) in each condition on skin conductance measures: Controlling for IQ**

| **Condition** | | | **ADHD case-control comparison** | | |  |
| --- | --- | --- | --- | --- | --- | --- |
|  |  | ***t*** | | ***p*** | ***Cohen’s d*** | |
| **Resting time 1** | *SCL* | -0.44 | | 0.66 | -0.07 | |
|  | *NSFs* | 0.50 | | 0.62 | 0.08 | |
| **CPT-OX** | *SCL* | 0.20 | | 0.84 | 0.03 | |
|  | *NSFs* | 0.40 | | 0.69 | 0.06 | |
| **Fast Task:**  **Baseline** | *SCL* | -1.17 | | 0.24 | -0.09 | |
|  | *NSFs* | 2.48 | | **0.01** | 0.38 | |
| **Fast Task:**  **Fast-Incentive** | *SCL* | 0.55 | | 0.58 | 0.10 | |
|  | *NSFs* | 1.26 | | 0.21 | 0.23 | |
| **Resting time 2** | *SCL* | 0.68 | | 0.50 | 0.10 | |
|  | *NSFs* | 2.37 | | **0.02** | 0.36 | |

*CPT-OX: Continuous performance task. SCL: Skin conductance level. NSF: non-specific fluctuations per second.*

**Table A.3: Main and interaction associations between skin conductance and IQ.**

|  | **NSFs** | | | | **SCL** | | | |
| --- | --- | --- | --- | --- | --- | --- | --- | --- |
|  | Main | | Interaction | | Main | | Interaction | |
| **IQ** | *Beta* | *p* | *Beta* | *p* | *Beta* | *p* | *Beta* | *p* |
| **Rest1** | 0.02 | 0.80 | 0.18 | 0.20 | **0.14** | **0.04** | 0.22 | 0.21 |
| **CPT-OX** | 0.05 | 0.48 | 0.17 | 0.27 | **0.17** | **0.02** | 0.30 | 0.09 |
| **Fast Task: Baseline** | 0.02 | 0.77 | -0.07 | 0.62 | **0.40** | **<0.01** | 0.10 | 0.49 |
| **Fast Task: Fast-Incentive** | **0.25** | **<0.01** | 0.15 | 0.48 | **0.26** | **<0.01** | **-0.15** | **<0.01** |
| **Rest 2** | -0.01 | 0.96 | 0.02 | 0.89 | **0.17** | **0.02** | 0.04 | 0.83 |

*CPT-OX: Continuous performance task. SCL: Skin conductance level. NSF: non-specific fluctuations per second.*

**Table A.4: Pair-wise tests between groups (ADHD and control) in each condition on skin conductance measures: Controlling for age and gender**

| **Condition** | | | **ADHD case-control comparison** | | | |
| --- | --- | --- | --- | --- | --- | --- |
|  |  | ***t*** | | ***df*** | ***p*** | ***Cohen’s d*** |
| **Rest time 1** | *SCL* | -1.31 | | 176 | 0.19 | -0.20 |
|  | *NSFs* | 0.27 | | 175 | 0.78 | 0.04 |
| **CPT-OX** | *SCL* | -0.90 | | 188 | 0.37 | -0.13 |
|  | *NSFs* | -0.11 | | 188 | 0.91 | 0.02 |
| **Fast Task:**  **Baseline** | *SCL* | -3.71 | | 171 | **<.01** | -0.57 |
|  | *NSFs* | 1.98 | | 173 | **0.05** | 0.30 |
| **Fast Task:**  **Fast-Incentive** | *SCL* | -1.40 | | 169 | 0.16 | -0.21 |
|  | *NSFs* | -0.78 | | 169 | 0.44 | -0.12 |
| **Rest time 2** | *SCL* | -0.39 | | 180 | 0.70 | -0.06 |
|  | *NSFs* | 2.04 | | 177 | **0.04** | 0.31 |

*CPT-OX: Continuous performance task. SCL: Skin conductance level. NSF: non-specific fluctuations per second.*

**Supplementary A.5: Results on the associations between IQ scores and skin conductance measures**

Linear regression models revealed significant (p < 0.05) and positive associations between skin conductance level and IQ (Beta = 0.17-0.40) in all five testing conditions. The interaction term (skin conductance level*group) was only significant in the Fast Task Fast-Incentive condition, revealing that associations were similar across groups for all conditions except for the Fast-Incentive condition. Overall, these findings show that individuals with lower levels of tonic arousal tended to have lower IQ scores. We found that the association between non-specific fluctuations and IQ was only significant in the Fast Task Fast-Incentive condition, suggesting that non-specific fluctuations were significantly related to higher IQ scores in this high-demanding task (Table A.3).


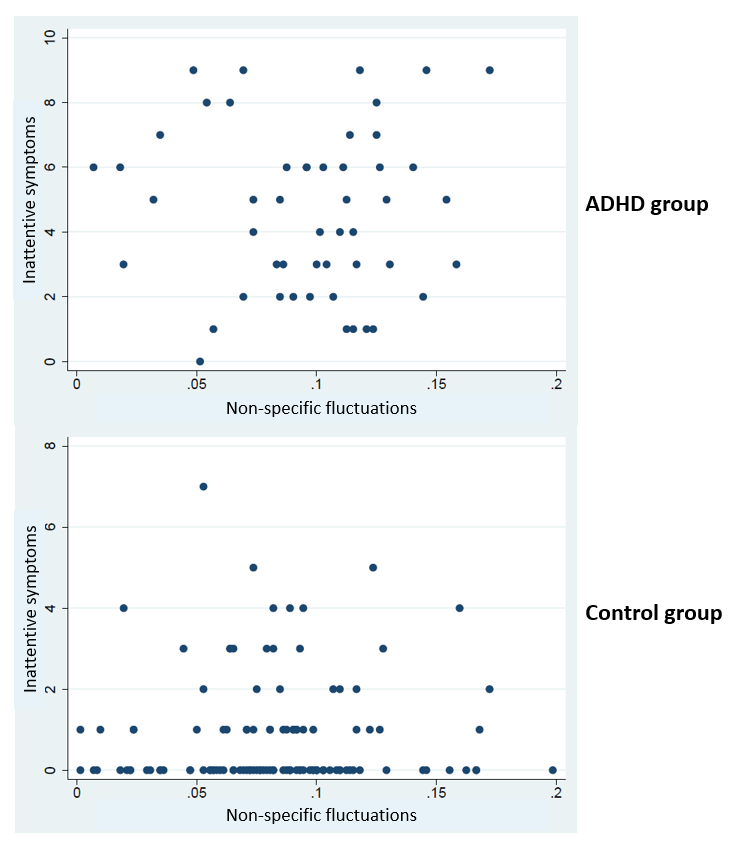


**Figure A.1: Scatterplots of non-specific fluctuations (during the Fast Task: Baseline) and inattentive symptoms in the ADHD and control groups**
